# Supplementary material for: Mevalonate Pathway Enzyme HMGCS1 Contributes to Gastric Cancer Progression
Source: Cancers (Basel). 2020 Apr 27;12(5):1088. doi: 10.3390/cancers12051088 (PMC7281414; doi:10.3390/cancers12051088)

Figure 1C

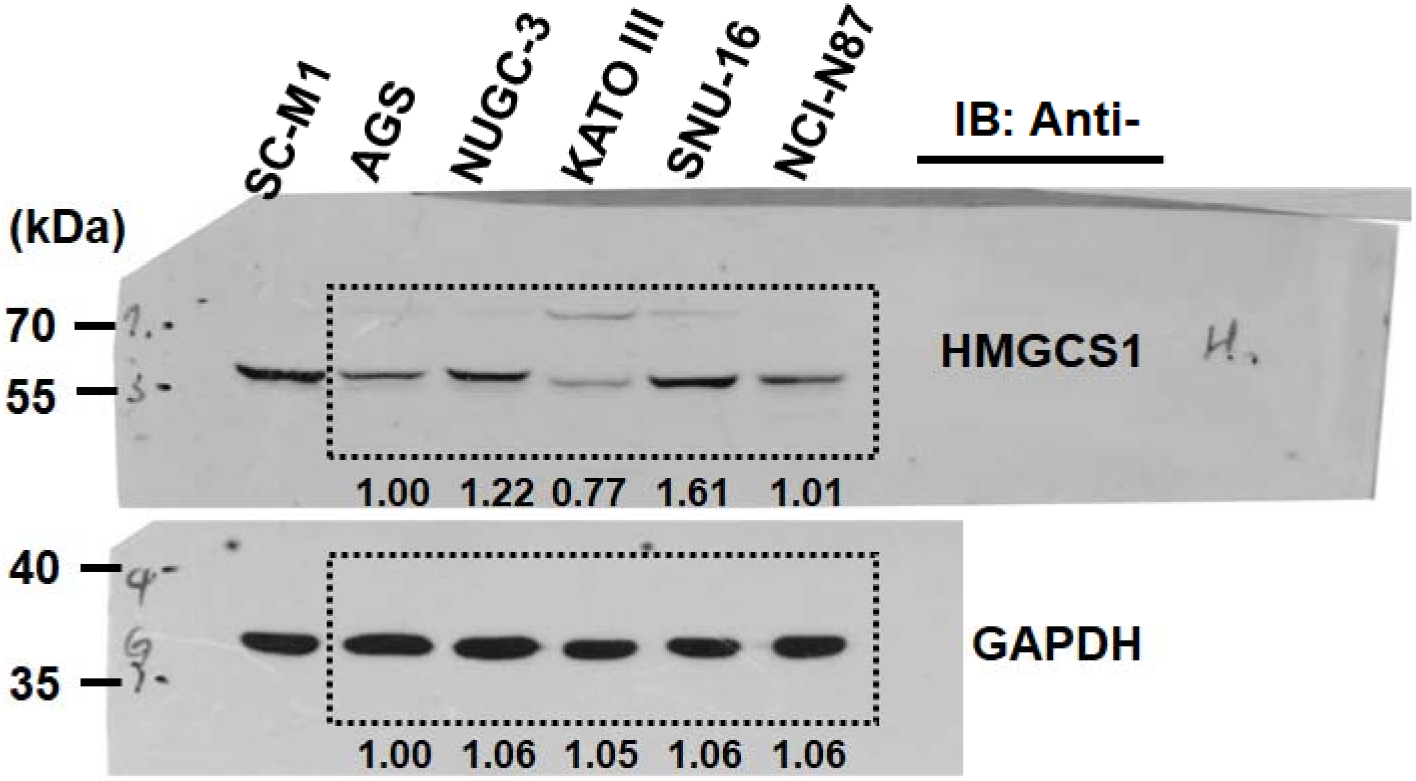
SC-M1
AGS
NUGC-3
KATO III
SNU-16
NCI-N87
IB: Anti-
(kDa)
70
55
HMGCS1
1.00 1.22 0.77 1.61 1.01
40
35
GAPDH
1.00 1.06 1.05 1.06 1.06

Figure 1E

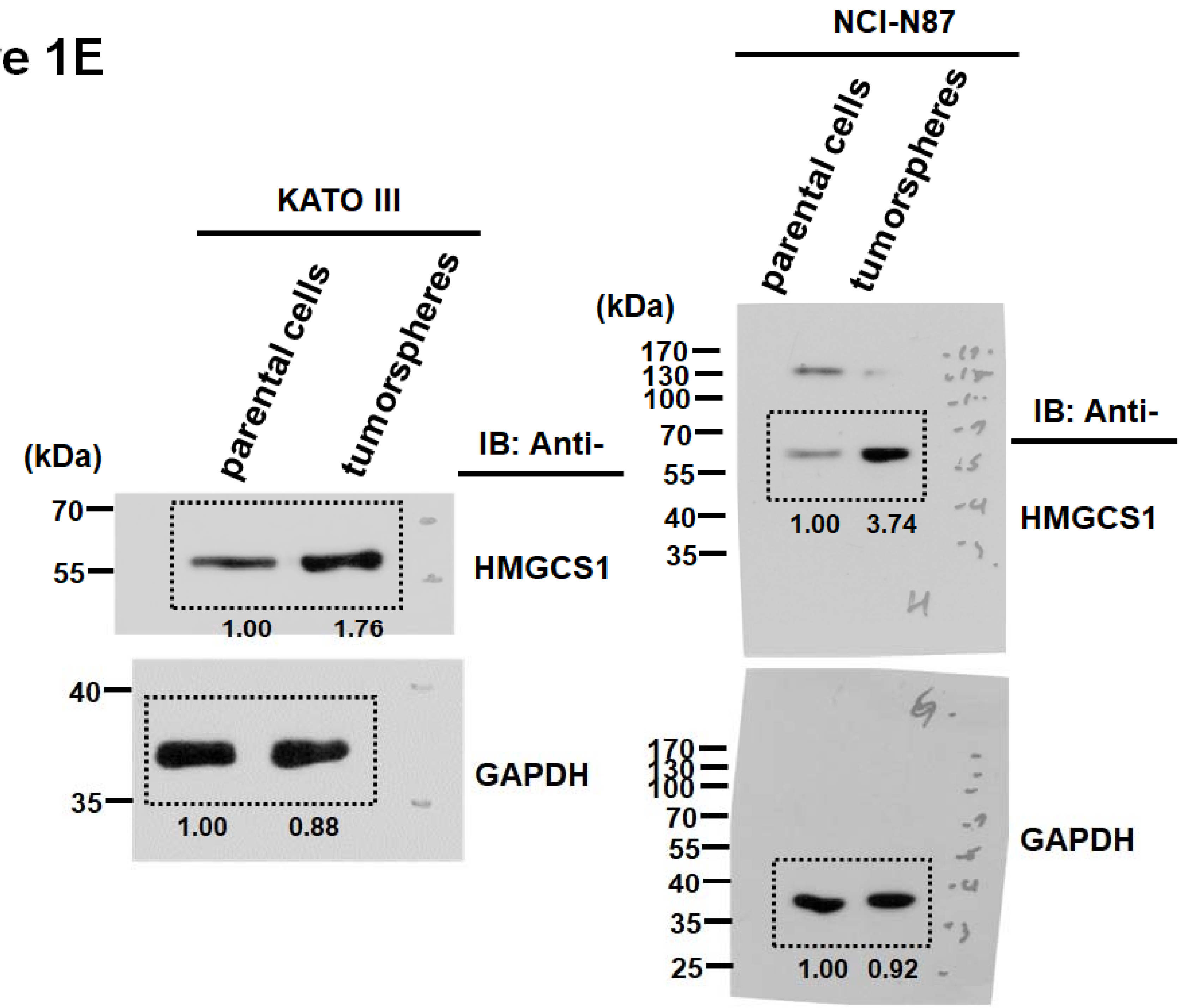
KATO III
parental cells
tumorspheres
(kDa)
70
55
HMGCS1
1.00
1.76
40
35
GAPDH
1.00
0.88
IB: Anti-
NCI-N87
parental cells
tumorspheres
(kDa)
170
130
100
70
55
40
35
HMGCS1
1.00
3.74
IB: Anti-
170
130
100
70
55
40
35
25
GAPDH
1.00
0.92

Figure 2B

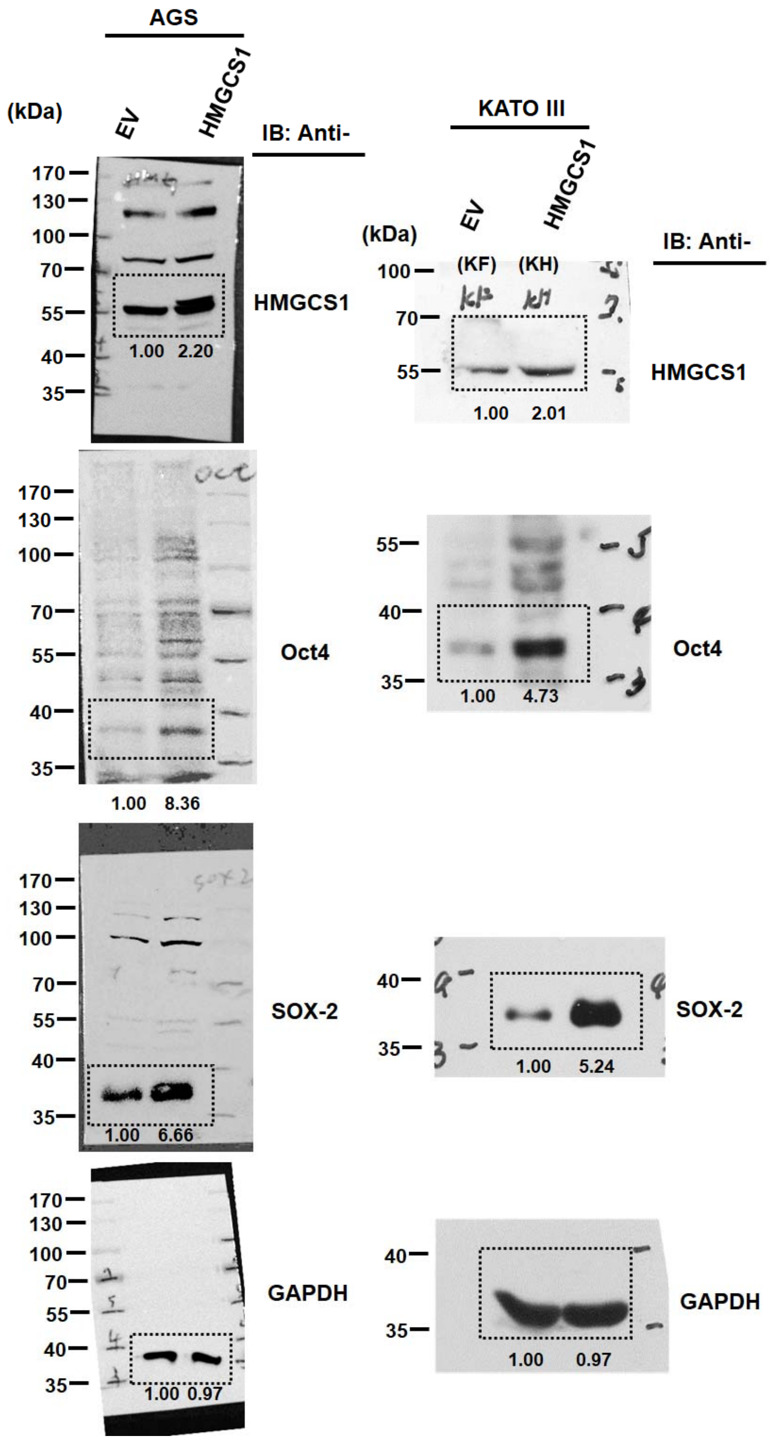
AGS
EV
HMGCS1
(kDa)
IB: Anti-
170
130
100
70
55
40
35
HMGCS1
1.00 2.20
Oct4
1.00 8.36
SOX-2
1.00 6.66
GAPDH
1.00 0.97
KATO III
EV
HMGCS1
(KF)
(KH)
1.00 2.01
1.00 4.73
1.00 5.24
1.00 0.97

Figure 4A

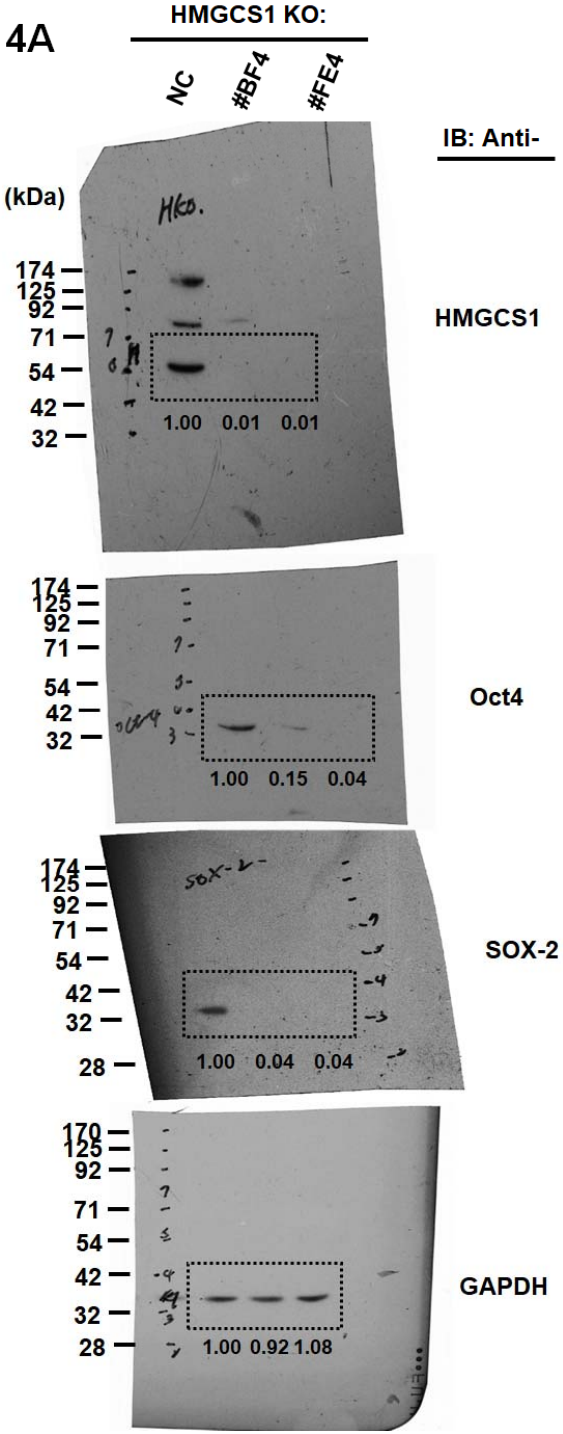
HMGCS1 KO:
NC
#BF4
#FE4
IB: Anti-
(kDa)
Hko.
174
125
92
71
54
42
32
HMGCS1
1.00 0.01 0.01
174
125
92
71
54
42
32
Oct4
1.00 0.15 0.04
174
125
92
71
54
42
32
28
SOX-2
1.00 0.04 0.04
170
125
92
71
54
42
32
28
GAPDH
1.00 0.92 1.08

Figure 5A

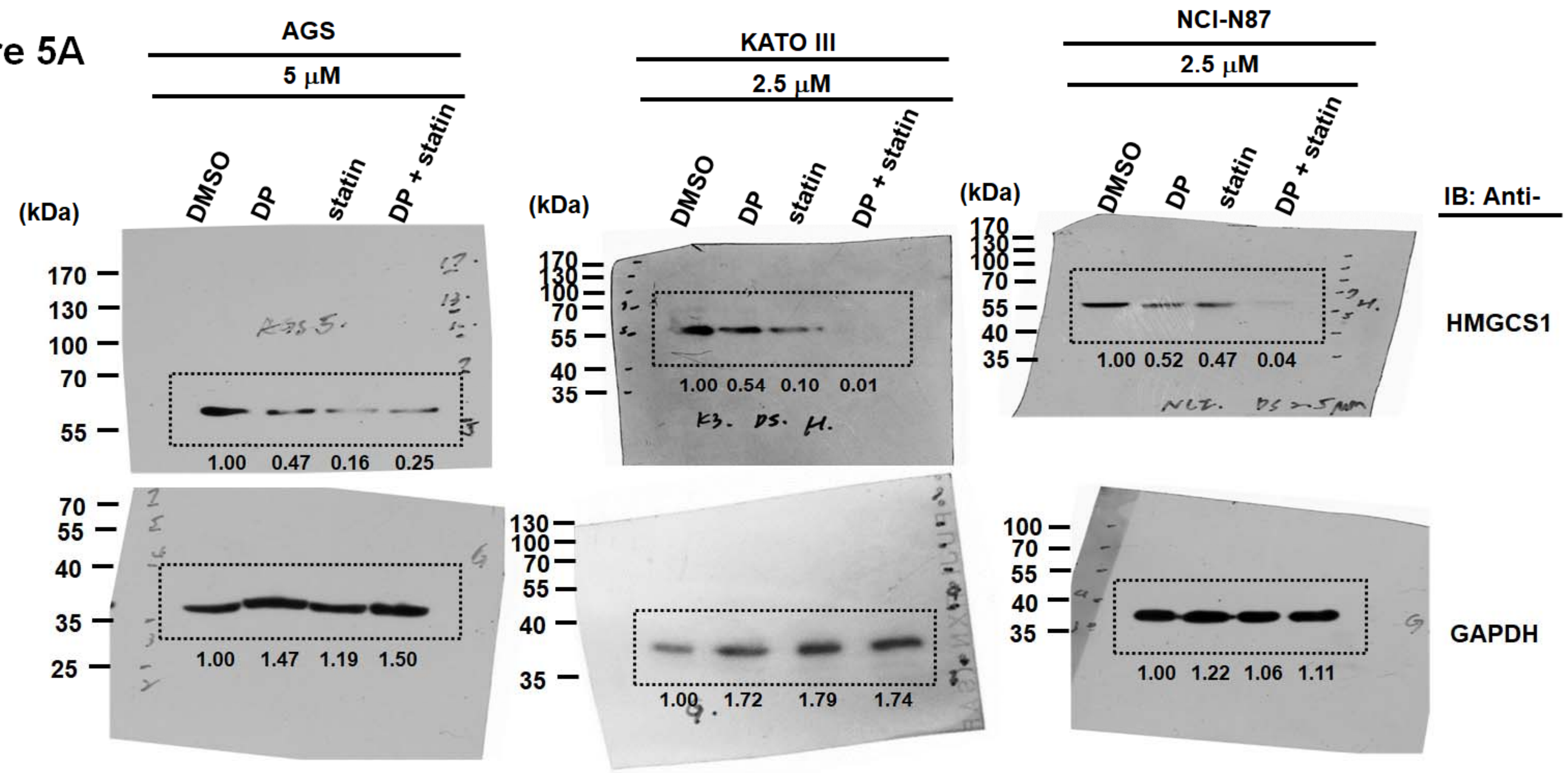
AGS
5 μM
KATO III
2.5 μM
NCI-N87
2.5 μM
DMSO
DP
statin
DP + statin
(kDa)
IB: Anti-
HMGCS1
GAPDH
1.00 0.47 0.16 0.25
1.00 0.54 0.10 0.01
1.00 0.52 0.47 0.04
1.00 1.47 1.19 1.50
1.00 1.72 1.79 1.74
1.00 1.22 1.06 1.11

Figure 6A

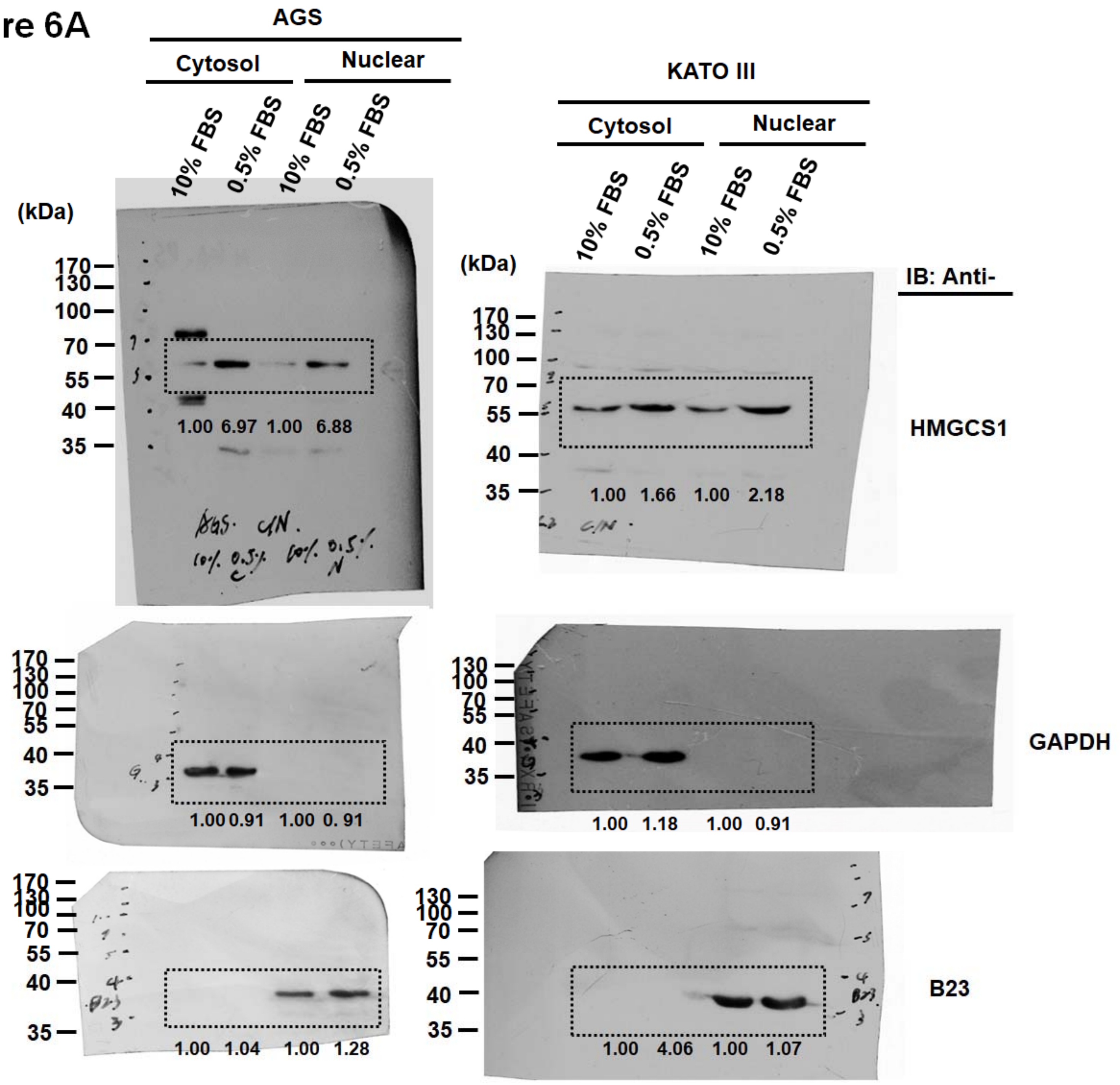

AGS
Cytosol
Nuclear
10% FBS
0.5% FBS
10% FBS
0.5% FBS
(kDa)
170
130
100
70
55
40
35
1.00 6.97 1.00 6.88
KATO III
Cytosol
Nuclear
IB: Anti-
HMGCS1
1.00 1.66 1.00 2.18
GAPDH
1.00 0.91 1.00 0. 91
1.00 1.18 1.00 0.91
B23
1.00 1.04 1.00 1.28
1.00 4.06 1.00 1.07

Figure 6B

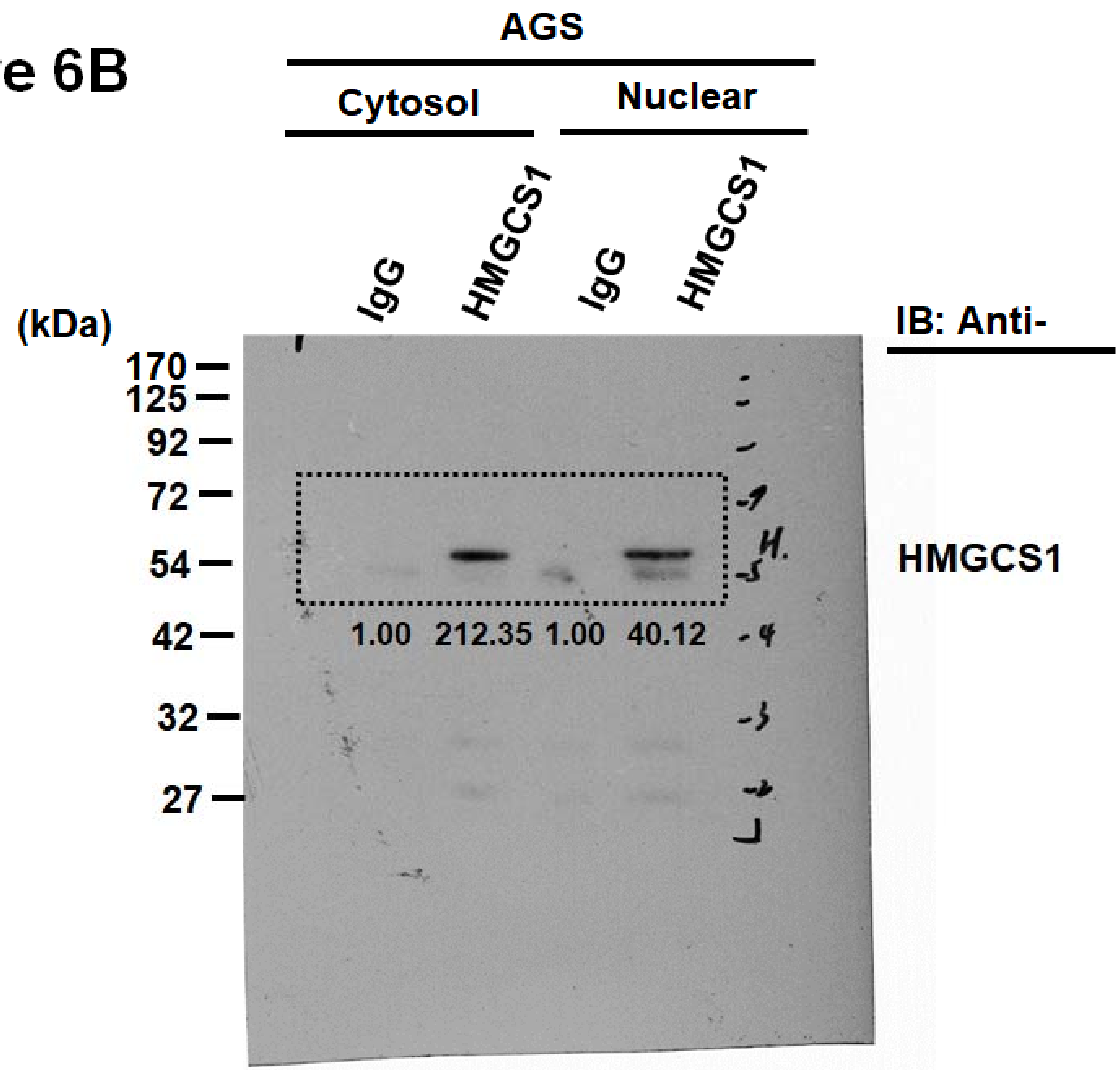
AGS
Cytosol
Nuclear
IgG
HMGCS1
IgG
HMGCS1
(kDa)
170
125
92
72
54
42
32
27
1.00
212.35
1.00
40.12
IB: Anti-
HMGCS1

Figure 6E

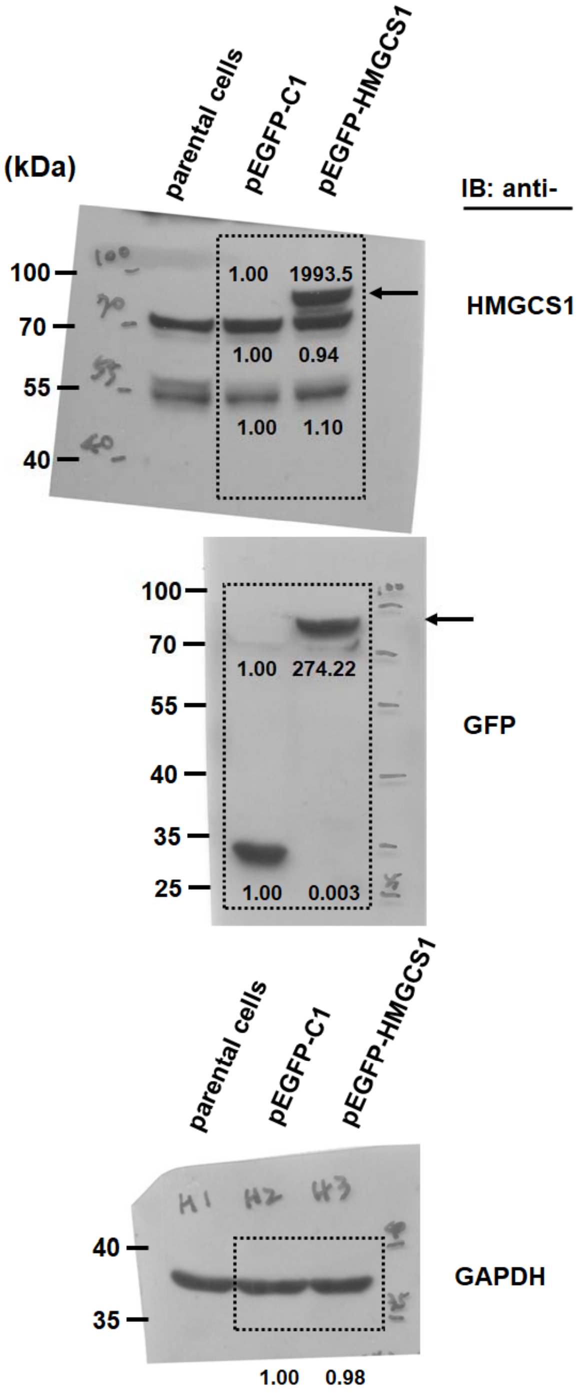
(kDa)
parental cells
pEGFP-C1
pEGFP-HMGCS1
IB: anti-
100
70
55
40
1.00
1993.5
1.00
0.94
1.00
1.10
HMGCS1
100
70
55
40
35
25
1.00
274.22
1.00
0.003
GFP
parental cells
pEGFP-C1
pEGFP-HMGCS1
40
35
GAPDH
1.00
0.98

Figure 7A

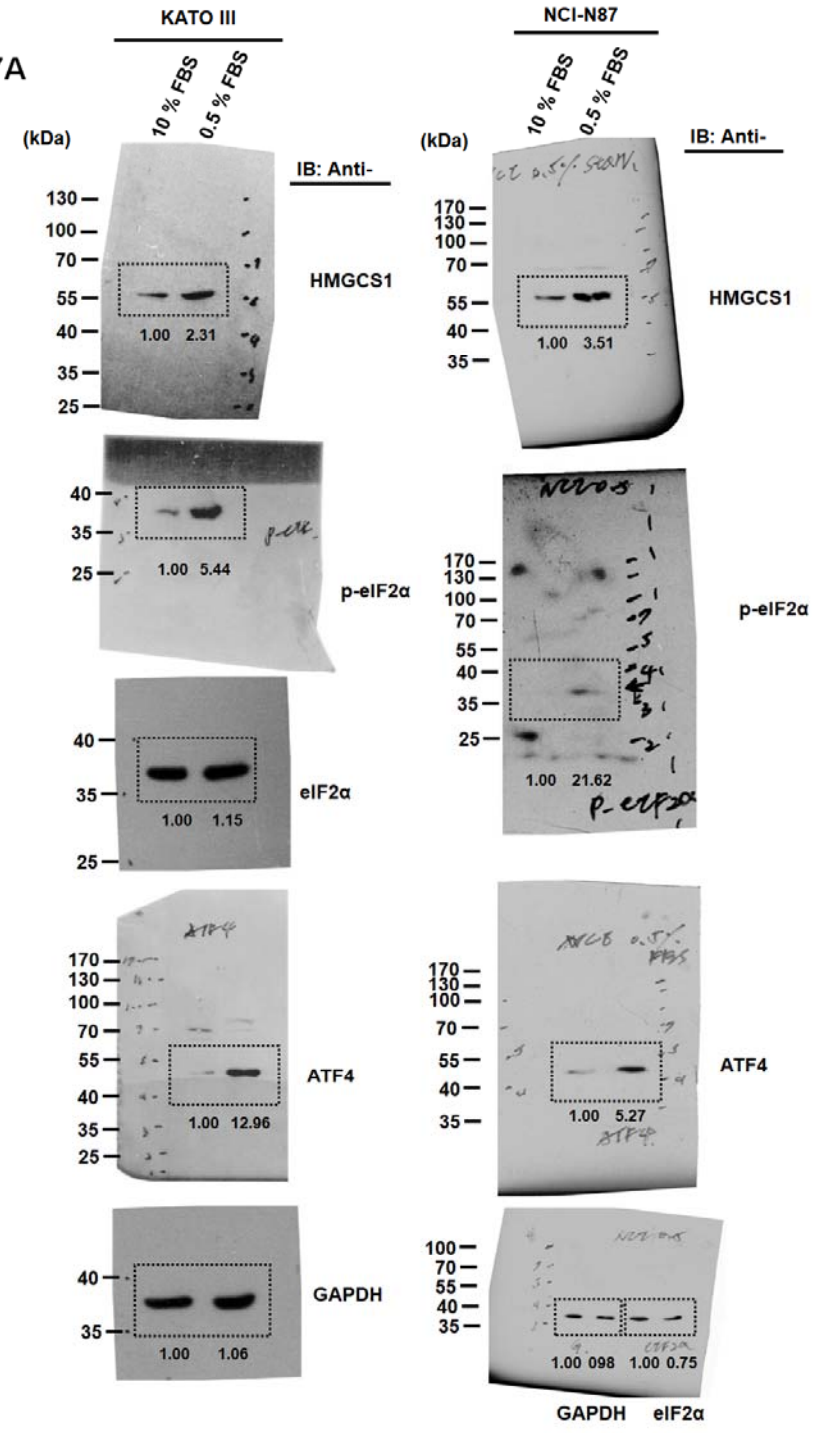
KATO III
10 % FBS
0.5 % FBS
(kDa)
IB: Anti-
HMGCS1
1.00 2.31
p-eIF2α
1.00 5.44
eIF2α
1.00 1.15
ATF4
1.00 12.96
GAPDH
1.00 1.06
NCI-N87
10 % FBS
0.5 % FBS
(kDa)
IB: Anti-
HMGCS1
1.00 3.51
p-eIF2α
1.00 21.62
ATF4
1.00 5.27
1.00 098 1.00 0.75
GAPDH eIF2α
130
100
70
55
40
35
25
170

Figure 7B

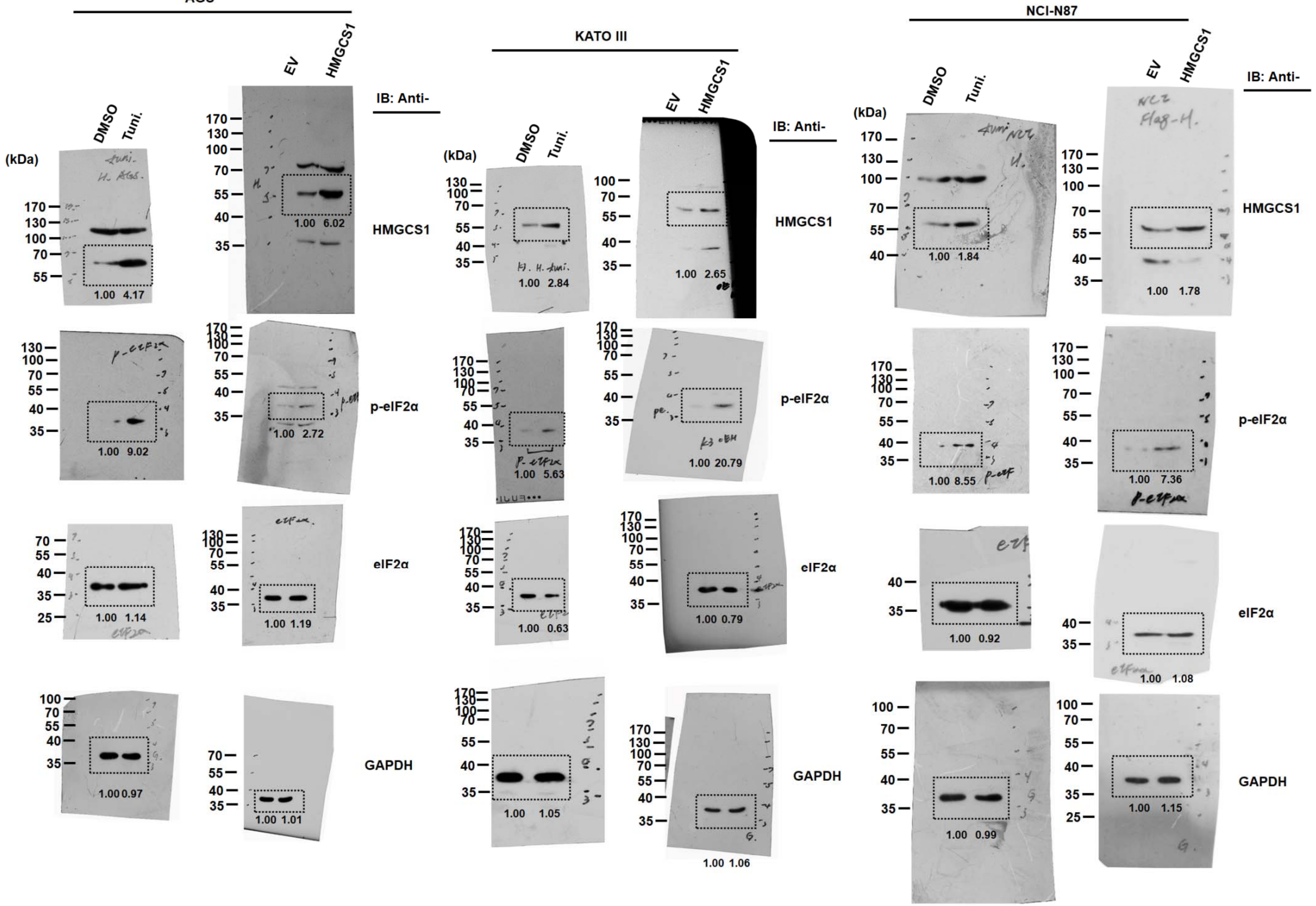
AGS
KATO III
NCI-N87
DMSO
Tuni.
EV
HMGCS1
IB: Anti-
(kDa)
HMGCS1
p-eIF2α
eIF2α
GAPDH
1.00 4.17
1.00 6.02
1.00 2.84
1.00 2.65
1.00 1.84
1.00 1.78
1.00 9.02
1.00 2.72
1.00 5.63
1.00 20.79
1.00 8.55
1.00 7.36
1.00 1.14
1.00 1.19
1.00 0.63
1.00 0.79
1.00 0.92
1.00 1.08
1.00 0.97
1.00 1.01
1.00 1.05
1.00 1.06
1.00 0.99
1.00 1.15

Figure 7C

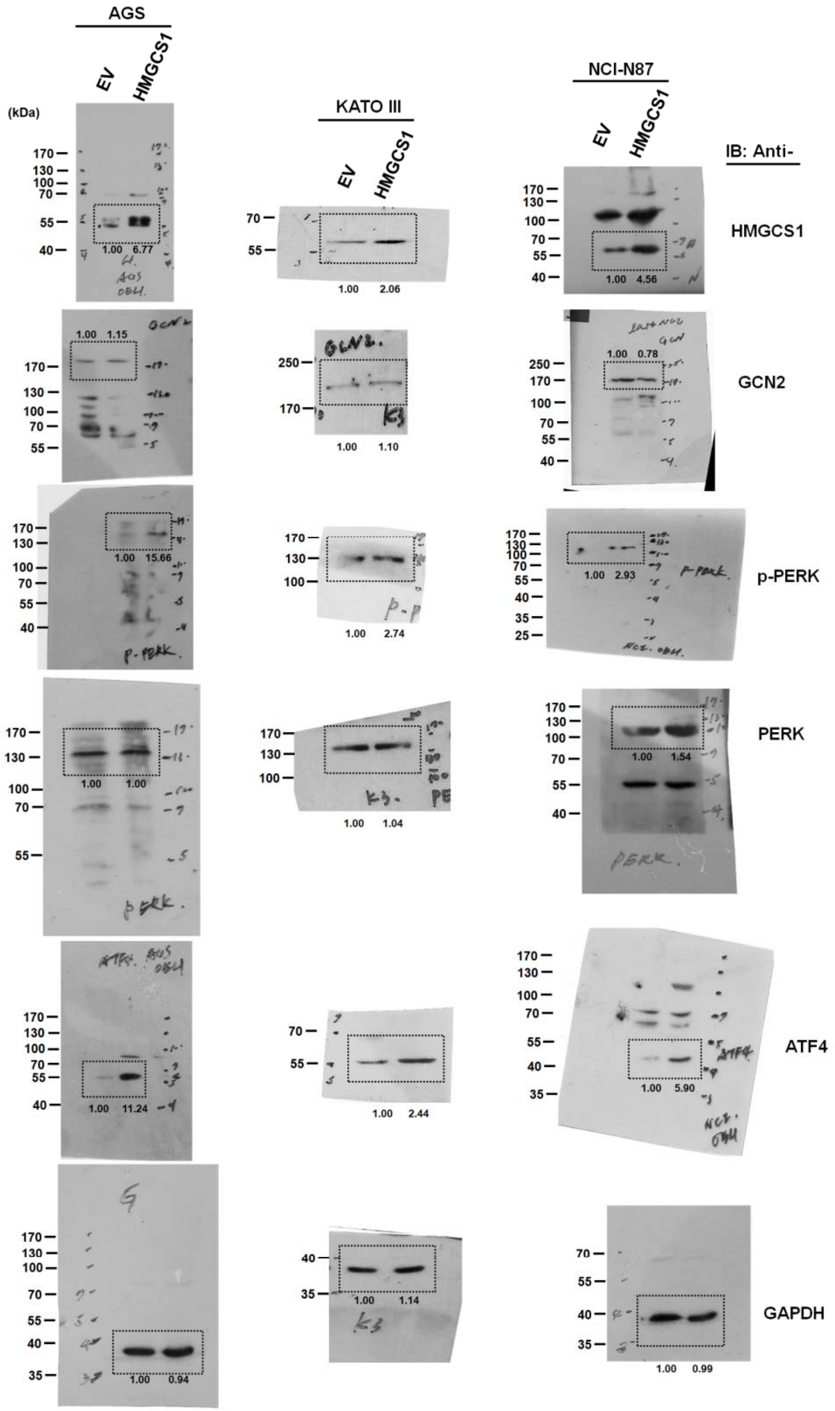
AGS
EV
HMGCS1
KATO III
NCI-N87
IB: Anti-
(kDa)
HMGCS1
GCN2
p-PERK
PERK
ATF4
GAPDH
1.00 6.77
1.00 2.06
1.00 4.56
1.00 1.15
1.00 1.10
1.00 0.78
1.00 15.66
1.00 2.74
1.00 2.93
1.00 1.00
1.00 1.04
1.00 1.54
1.00 11.24
1.00 2.44
1.00 5.90
1.00 0.94
1.00 1.14
1.00 0.99

Figure 7E

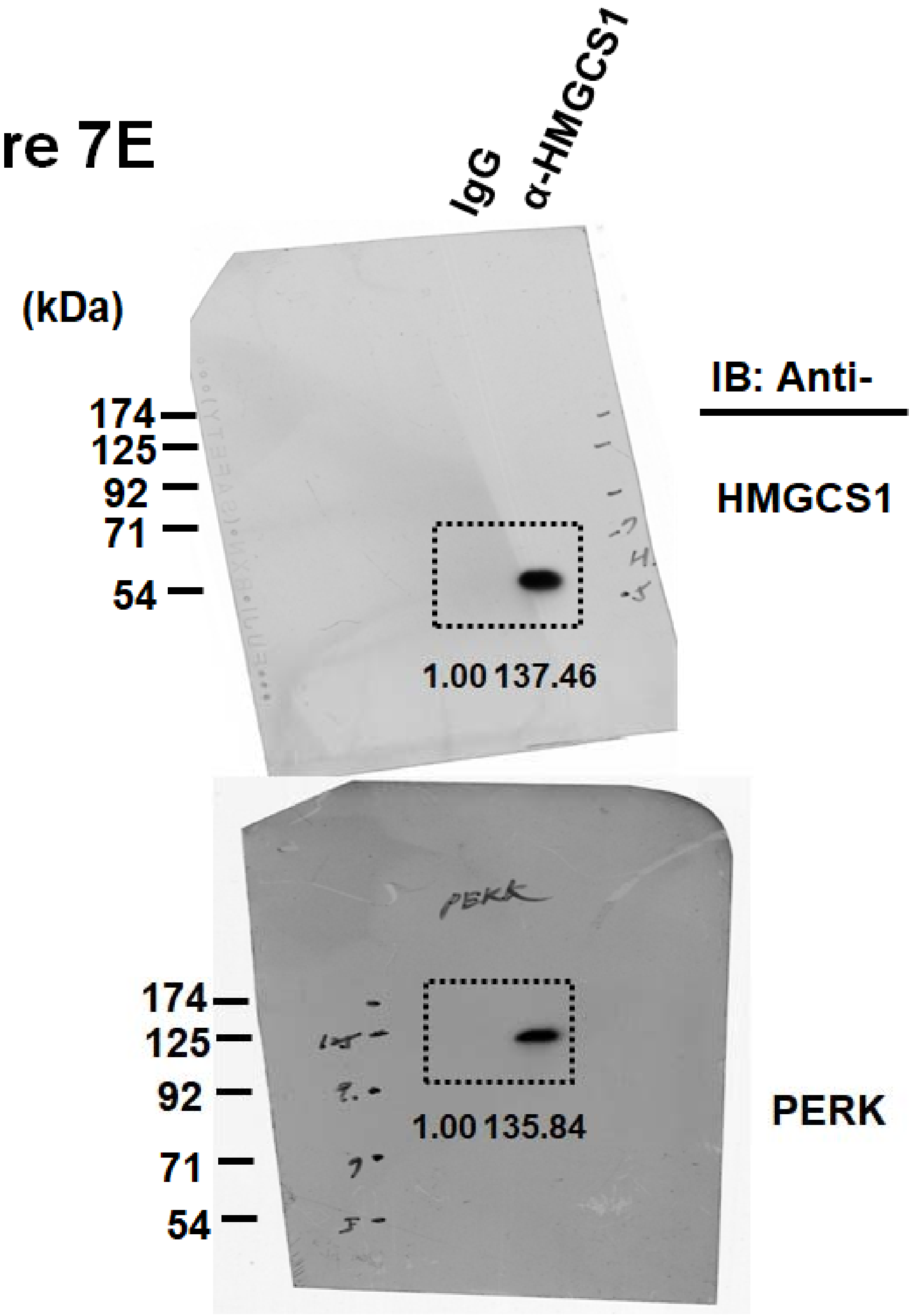
IgG
α-HMGCS1
(kDa)
174
125
92
71
54
1.00 137.46
IB: Anti-
HMGCS1
174
125
92
71
54
1.00 135.84
PERK

Supplementary Figure S2B

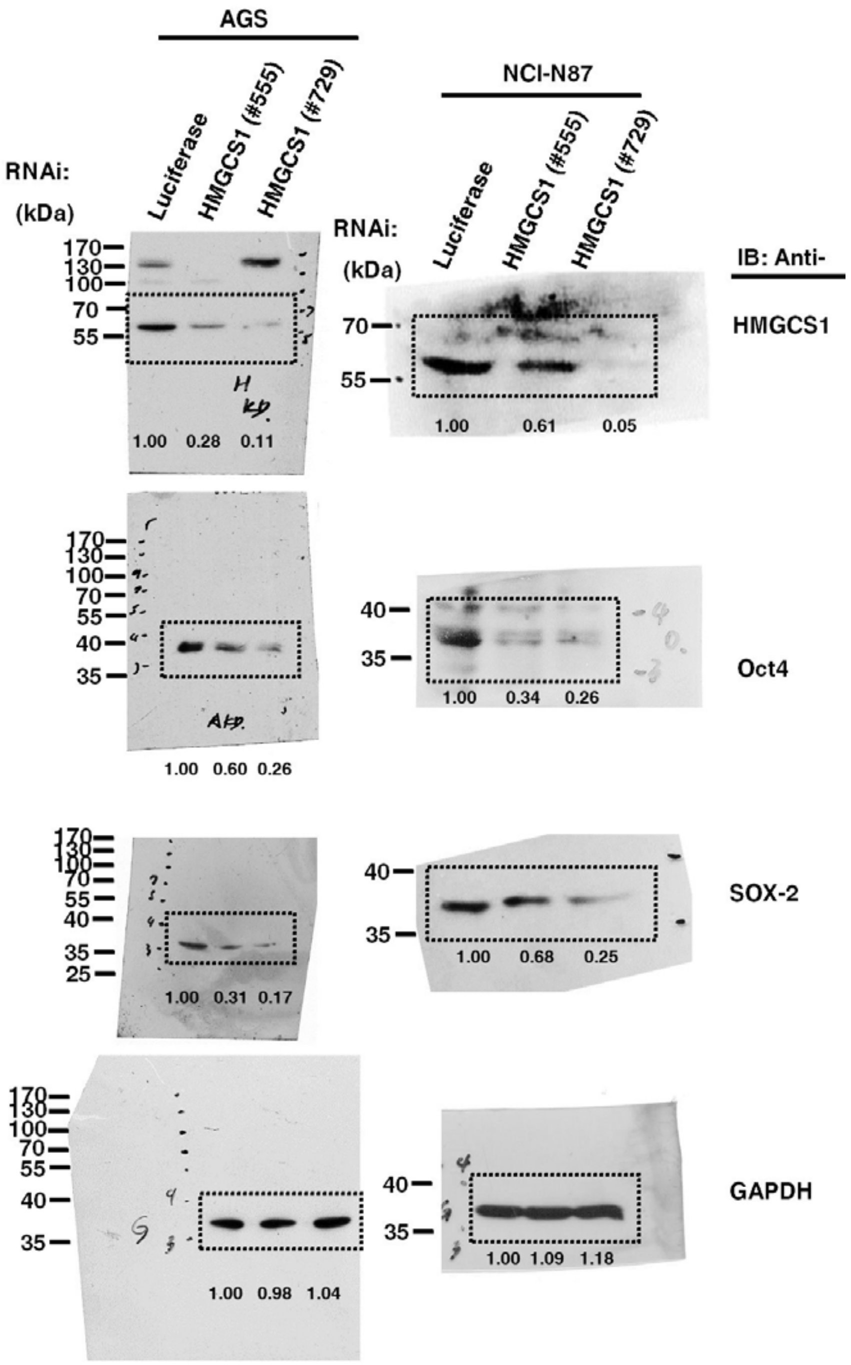

Supplementary Figure S5B

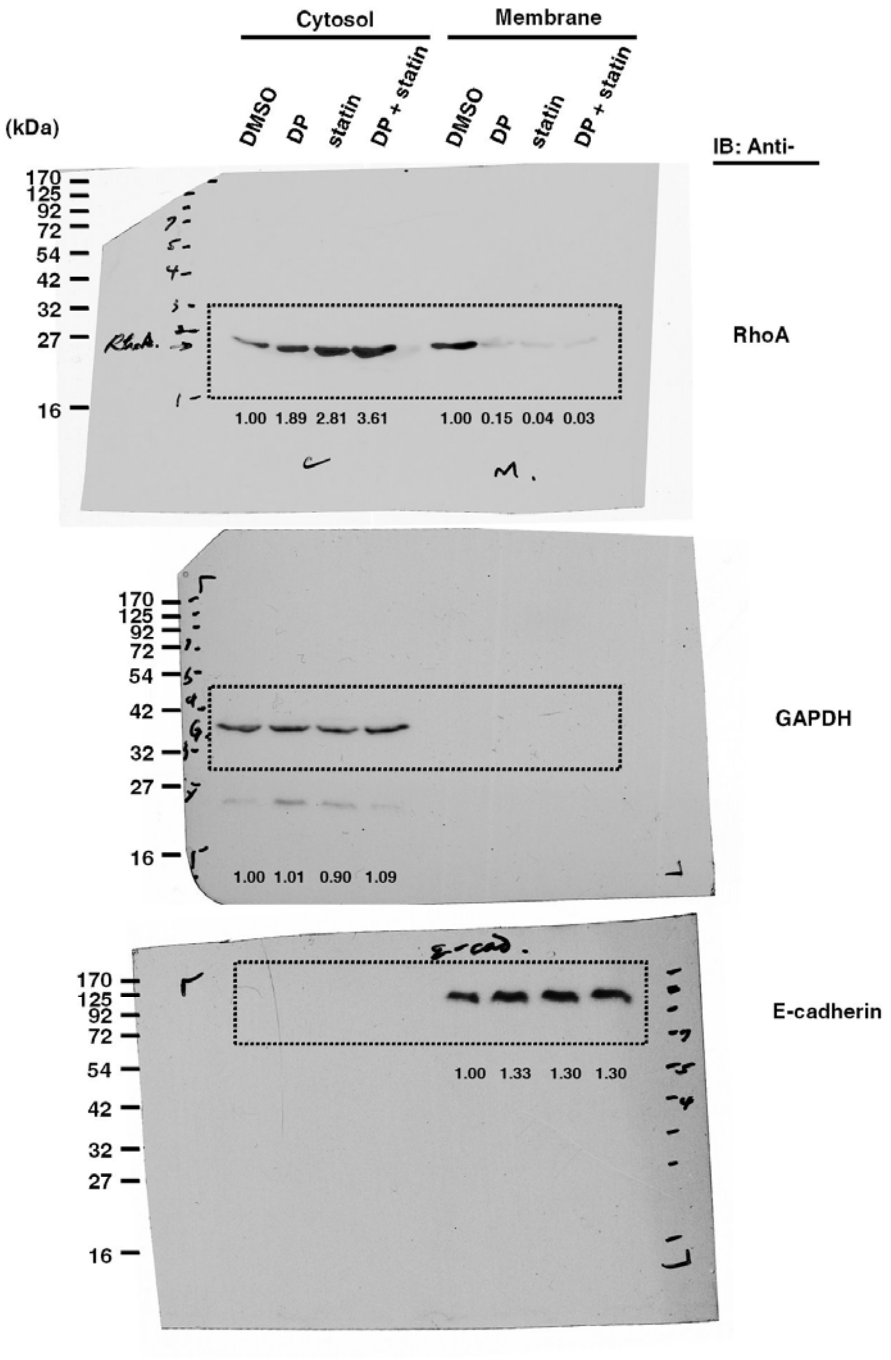

Supplement: Supplementary file 1 [file cancers-12-01088-s001.zip › Whole Western Blots-revised.pdf]
