# Supplementary material for: Mevalonate Pathway Enzyme HMGCS1 Contributes to Gastric Cancer Progression
Source: Cancers (Basel). 2020 Apr 27;12(5):1088. doi: 10.3390/cancers12051088 (PMC7281414; doi:10.3390/cancers12051088)
Supplement: Supplementary file 1 [file cancers-12-01088-s001.zip › cancers-755403 - suppl.docx]

Article

Mevalonate Pathway Enzyme HMGCS1 Contributes to Gastric Cancer Progression

I-Han Wang ^1,2^, Tzu-Ting Huang ^2,3,†^, Ji-Lin Chen ^2,4,†^, Li-Wei Chu ^5,†^, Yueh-Hsin Ping ^5^,
Kai-Wen Hsu ^6,7^, Kuo-Hung Huang ^8,9^, Wen-Liang Fang ^8,9^, Hsin-Chen Lee ^5^, Chian-Feng Chen ^10^, Chen-Chung Liao ^11^, Rong-Hong Hsieh ^12^ and Tien-Shun Yeh ^2,10,13,14,^*

Supplementary Material

**Table S1.** Sequences of primers for siRNA, knockout, PCR, and real-time PCR.

| **Assays** |  | | **Sequence (5′ to 3′)** | **Amplicon (bp)** | | |
| --- | --- | --- | --- | --- | --- | --- |
| **siRNA**  **knockout**  **PCR**  **real-time PCR**  **ChIP** | **HMGCS1 (#555)**  **HMGCS1 (#729)**  **HMGCS1**  **HMGCS1 genomic DNA**  **HMGCS1**  **Oct4**  **SOX-2**  **XBP1s**  **XBP1u**  **total XBP1**  **ATF4**  **CHOP**  **GAPDH**  **Oct4 promoter**  **Oct4 coding sequence**  **SOX-2 promoter**  **SOX-2 coding sequence** | **TGTTGCCCTTGAGATCTATTT**  **GATCTTTCACTCACCATATTG**  **F CACCGGCTTGCTGGCCAAAAGATGT**  **R AAACACATCTTTTGGCCAGCAAGCC**  **F ATCTAGAGATGCCCAATTTTGAAT**  **R ATGATTGTCTCTGTTCCAACTT**  **F TGTACACATCTTCAGTATATGGTTCCC**  **R AAGAAAACACTCCAATTCTCTTCCCT**  **F GGGTGGAGGAAGCTGACAACAA**  **R AAATTCTCCAGGTTGCCTCTCA**  **F GTATCAGGAGTTGTCAGGCAGAG**  **R TCCTAGTCTTAAAGCGGCAGCAAAGC**  **F CTGAGTCCGCAGCAGGTGCAG**  **R ATCCATGGGGAGATGTTCTGG**  **F CAGCACTCAGACTACGTGCA**  **R ATCCATGGGGAGATGTTCTGG**  **F TGGCCGGGTGTGCTGAGTCCG**  **R ATCCATGGGGAGATGTTCTGG**  **F GTTCTCCAGCGACAAGGCTA**  **R ATCCTCCTTGCTGTTGTTGG**  **F AGAACCAGGAAACGGAAACAGA**  **R TCTCCTTCATGCGCTGCTTT**  **F AAATCCCATCACCATCTTCC**  **R TCACACCCATGACGAACA**  **F CCCTGGGGGTTCTATTTGGTGG**  **R ACTCCTCTTCATGGGTGAGGGT**  **F GGGTGGAGGAAGCTGACAACAA**  **R AGCTACGAGCCAGTGATGGAA**  **F GGATGGTTGTCTATTAACTTGT**  **R CTTCTTTCTCTCAGTCCTAGTC**  **F GTACAACATGATGGAGACGGAGC**  **R ATGAAGGCATTCATGGGCC** | | | **552**  **93**  **123**  **78**  **59**  **76**  **97**  **88**  **67**  **194**  **123**  **117**  **122**  **144** |  |


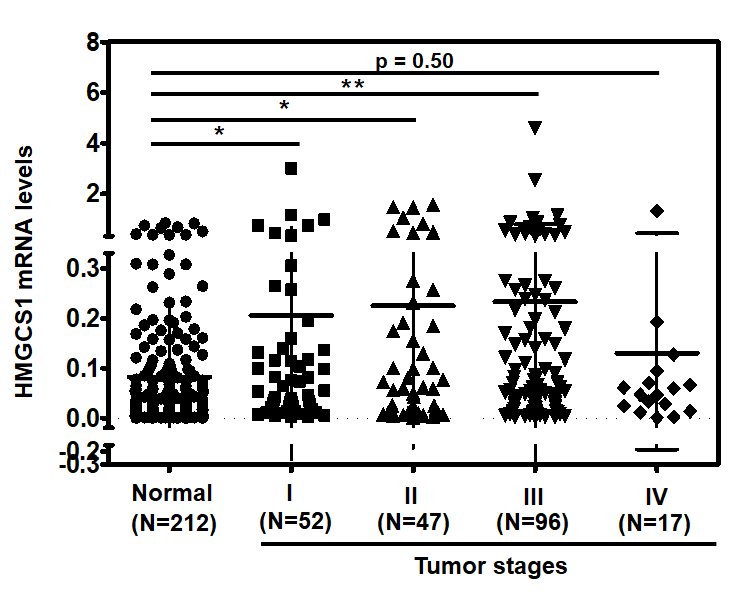


**Figure S1.** Expressions of HMGCS1 mRNA are increased in stomach adenocarcinoma specimens from patients with advanced stages of gastric cancer. The transcript levels of HMGCS1 in stomach adenocarcinoma samples from patients were divided according to the stage classification. Then levels of HMGCS1 mRNA in the gastric cancer tissues of stages I to IV (*n* = 212) were compared with those of the adjacent non-tumor tissues (*n* = 212). Mean ± SD. *, *P* < 0.05; **, *P* < 0.01.

**

**

**Figure S2.** HMGCS1 knockdown down-regulates levels of pluripotency genes Oct4 and SOX-2 and suppresses abilities of progression in gastric cancer cells. (**A,B**) NCI-N87 or AGS cells were infected with lentiviruses expressing siRNAs against HMGCS1 (#555 and #729) or luciferase for 48 hours. The transcript levels of Oct4 and SOX-2 in the infected NCI-N87 cells were determined by quantitative real-time PCR (A). Mean ± SD (*n* = 3). Whole-cell extracts of the infected AGS and NCI-N87 cells were prepared for Western blot analysis using anti-HMGCS1, anti-Oct4, anti-SOX-2, and anti-GAPDH antibodies (B). (**C,D**) NCI-N87 cells infected with lentiviruses expressing siRNAs against HMGCS1 (#555 and #729) or luciferase for 48 hours were seeded for assays of tumorsphere formation (C) and trypan blue exclusion (D). Mean ± SD (n=3). (**E**) AGS and NCI-N87 cells infected with lentiviruses expressing siRNAs against HMGCS1 or luciferase were seeded for MTT assay. Mean ± SD (*n* = 3). (**F**) The transfected NCI-N87 cells from (E) were seed for colony formation assay. Mean ± SD (*n* = 3). (**G**) The transfected cells from (E) were also used for migration (*upper*) and invasion (*lower*) assays. Mean ± SD (*n* = 3). *, *P* < 0.05; **, *P* < 0.01; ***, *P* < 0.001.


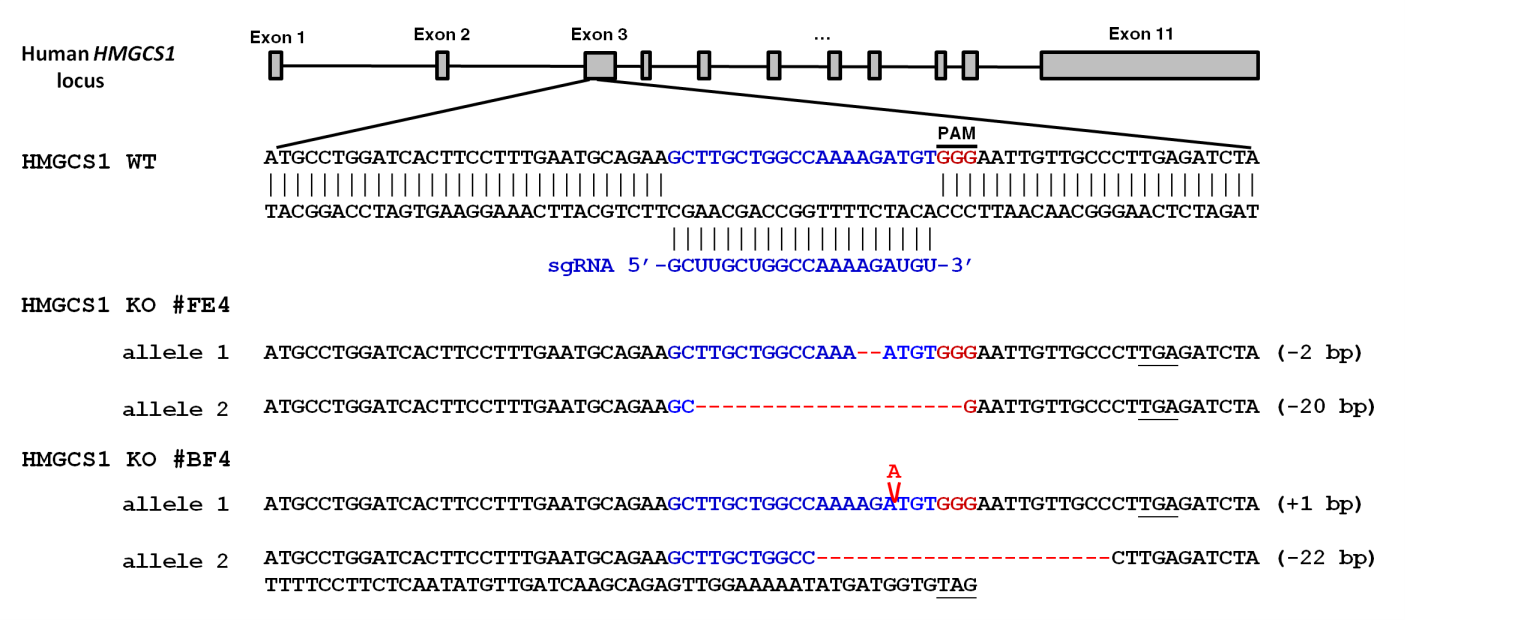


**Figure S3.** Schematic diagram of CRISPR-Cas9-mediated HMGCS1 knockout showing sequences of the sgRNA and PAM in wild type AGS cells as well as those of indels in knockout cells. Two clones of AGS cells with CRISPR-Cas9-mediated HMGCS1 knockout (HMGCS1 KO #FE4 and #BF4 cells) were established and their genotypes of two HMGCS1 alleles were validated by DNA sequencing. In these mutant clones, the introduced premature stop codons within the open reading frame of HMGCS1 were underlined.


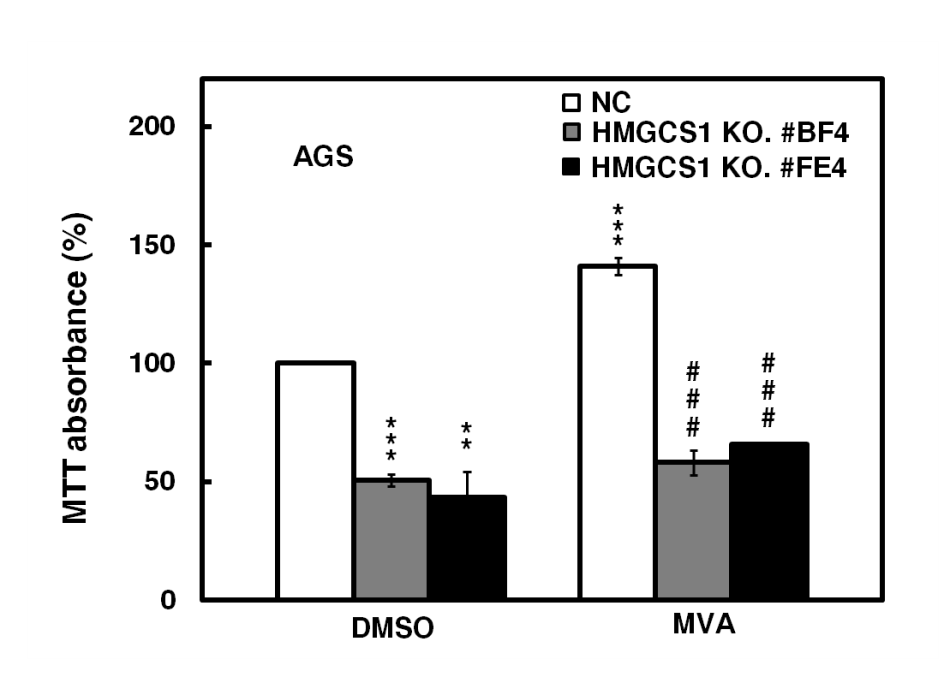


**Figure S4.** HMGCS1 knockout suppresses mevalonate-promoted ability of cell growth in AGS cells. HMGCS1 KO #BF4 and #FE4 cells and negative control (NC) cells were treated with 0.5 mM mevalonolactone (MVA) or an equal volume of DMSO for 24 hours for subsequent MTT assay. Mean ± SD (*n* = 3). **, *P* < 0.01; ***, *P* < 0.001. ###, *P* < 0.001.


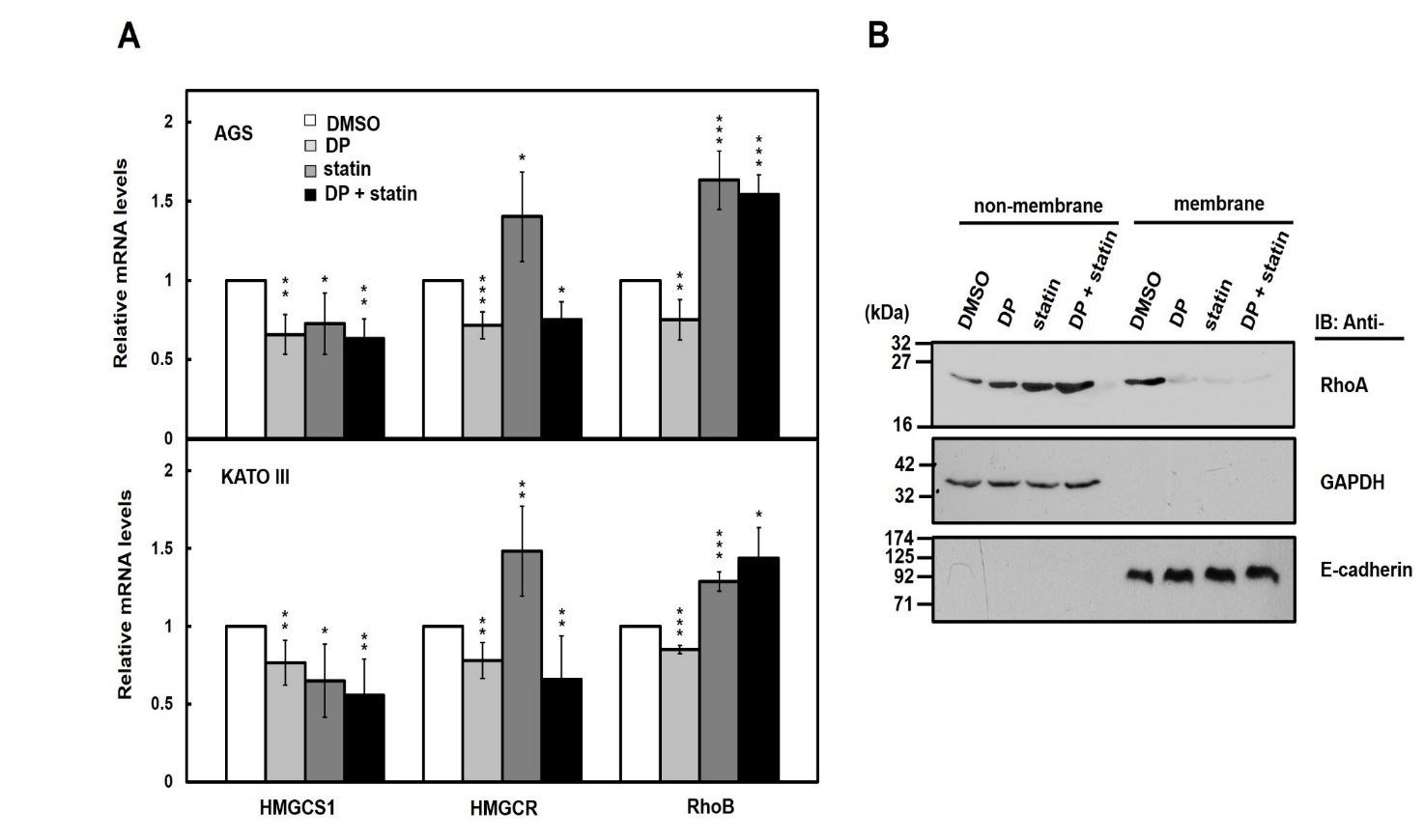


**Figure S5.** Co-treatment with lovastatin and dipyridamole blocks mevalonate pathway in gastric cancer cells. To block mevalonate pathway, AGS and KATO III cells were treated with 2.5 or 5 μM lovastatin (statin) and/or dipyridamole (DP) for 24 hours. (**A**) The transcript levels of HMGCS1, HMGCR, and RhoB in the treated cells were measured by quantitative real-time PCR and then normalized to GAPDH. Mean ± SD (*n* = 3). *, *P* < 0.05; **, *P* < 0.01; ***, *P* < 0.001. (**B**) Both membrane and non-membrane fractions of AGS cells treated with lovastatin and dipyridamole were also prepared for Western blot analysis using antibodies against RhoA, GAPDH (a marker of non-membrane fractions), and E-cadherin (a marker of membrane fractions).


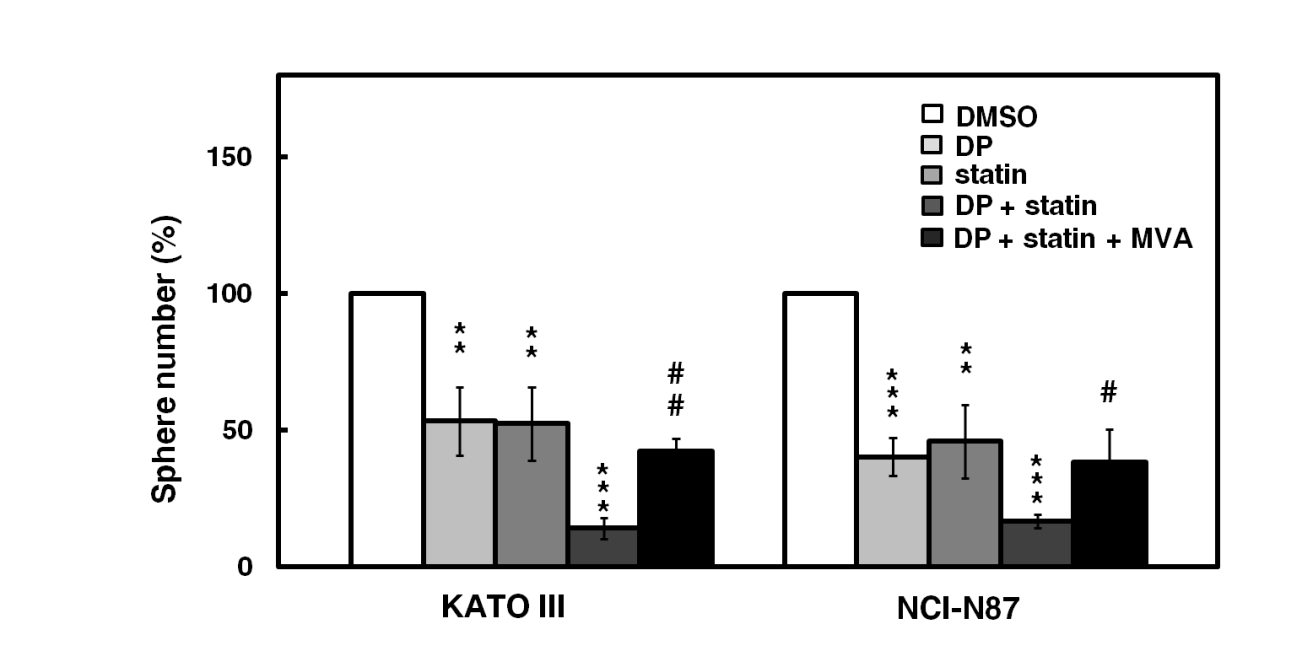


**Figure S6.** The statin- or dipyridamole-suppressed abilities of tumorsphere formation in KATO III and NCI-N87 cells are partially restored after mevalonate treatment. KATO III and NCI-N87 cells were treated with 2.5 μM of lovastatin (statin), 2.5 μM of dipyridamole (DP), and/or 0.5 mM mevalonolactone (MVA) for 24 hours and then seeded for the subsequent tumorsphere formation assay. Mean ± SD (*n* = 3). **, *P* < 0.01; ***, *P* < 0.001. #, *P* < 0.05; ##, *P* < 0.01.


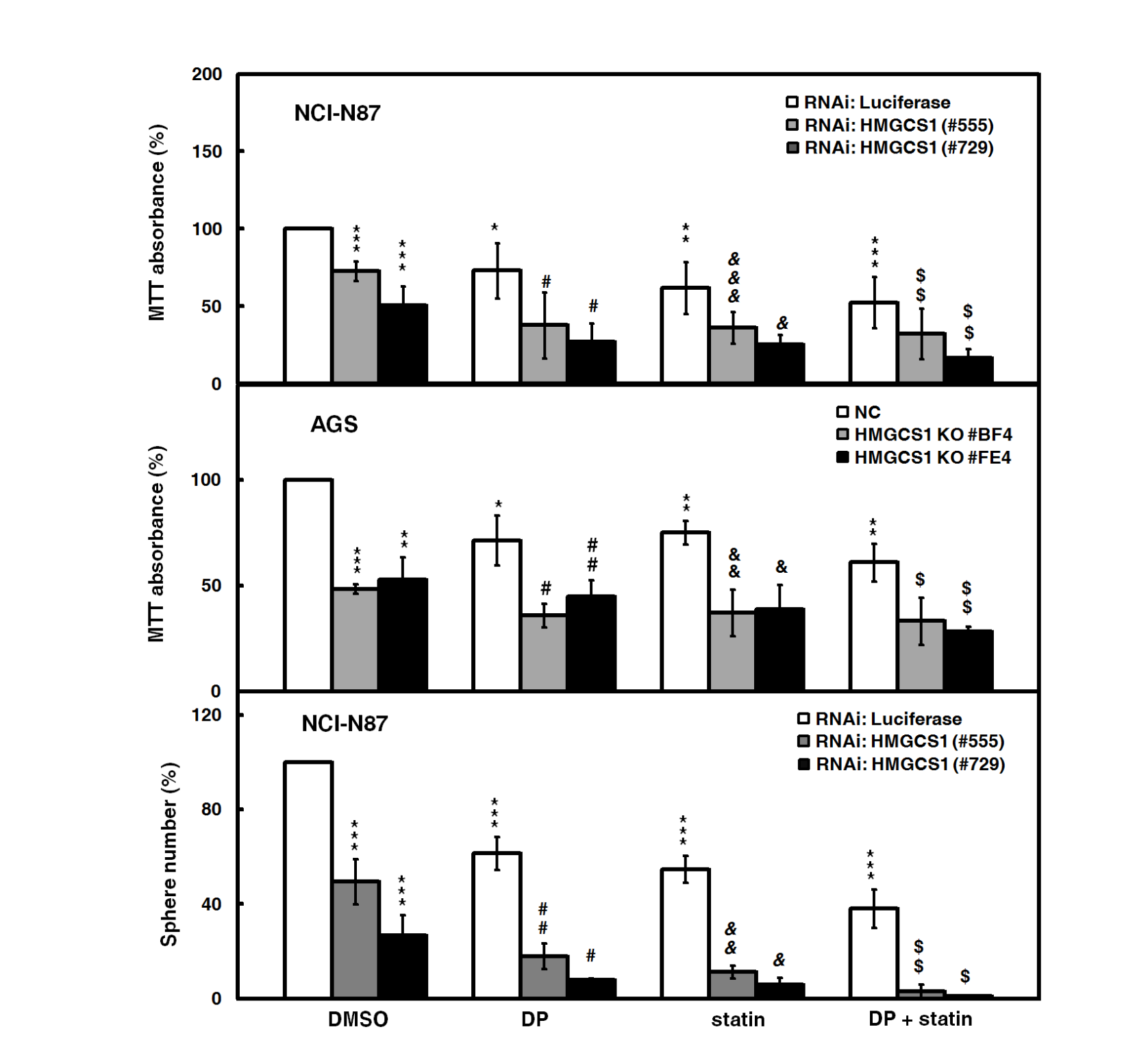


**Figure S7.** The statin- or dipyridamole-mediated decline of cell growth or tumorsphere formation abilities are further deteriorated in NCI-N87 cells with HMGCS1 knockdown and AGS cells with HMGCS1 knockout. NCI-N87 cells were infected with lentiviruses expressing siRNAs against HMGCS1 (#555 and #729) or luciferase for 48 hours. The infected NCI-N87 cells (*upper*) and HMGCS1 KO #BF4 and #FE4 cells (*middle*) were treated with 2.5 μM of lovastatin (statin) and/or dipyridamole (DP) for 24 hours for subsequent MTT assay. The infected NCI-N87 cells were also seeded for tumorsphere formation assay in the presence or absence of 2.5 μM of lovastatin and/or dipyridamole (*lower*). Mean ± SD (*n* = 3). *, *P* < 0.05; **, *P* < 0.01; ***, *P* < 0.001. #, *P* < 0.05; ##, *P* < 0.01. &, *P* < 0.05; &&, *P* < 0.01; &&&, *P* < 0.001. $, *P* < 0.05; $$, *P* < 0.01.


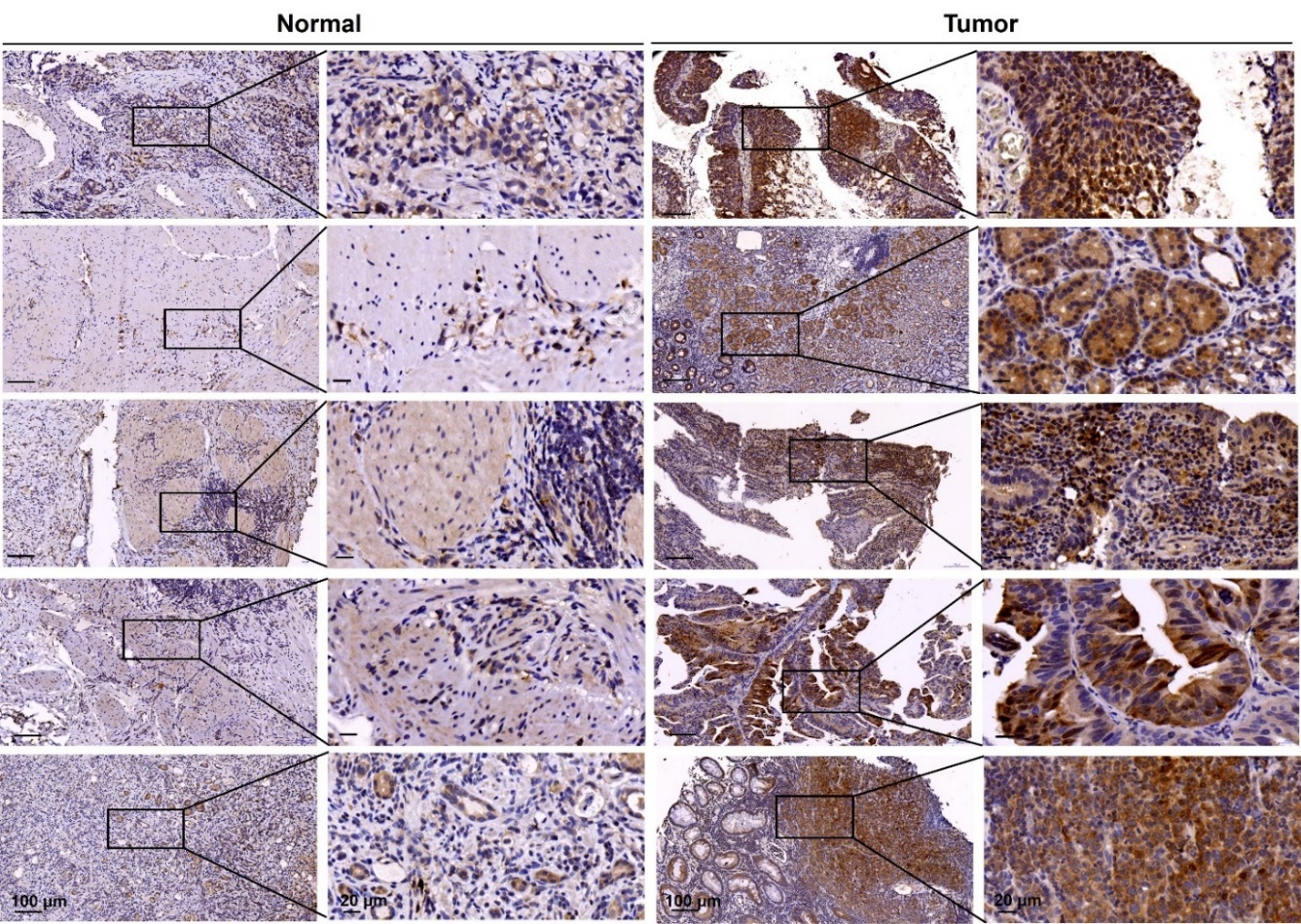


**Figure S8**. HMGCS1 is present in both nuclei and cytoplasms of gastric cancer tissues from patients. HMGCS1 expressions in tumors and the adjacent normal tissues from gastric cancer patients were examined by immunohistochemistry analysis using antibody against HMGCS1.


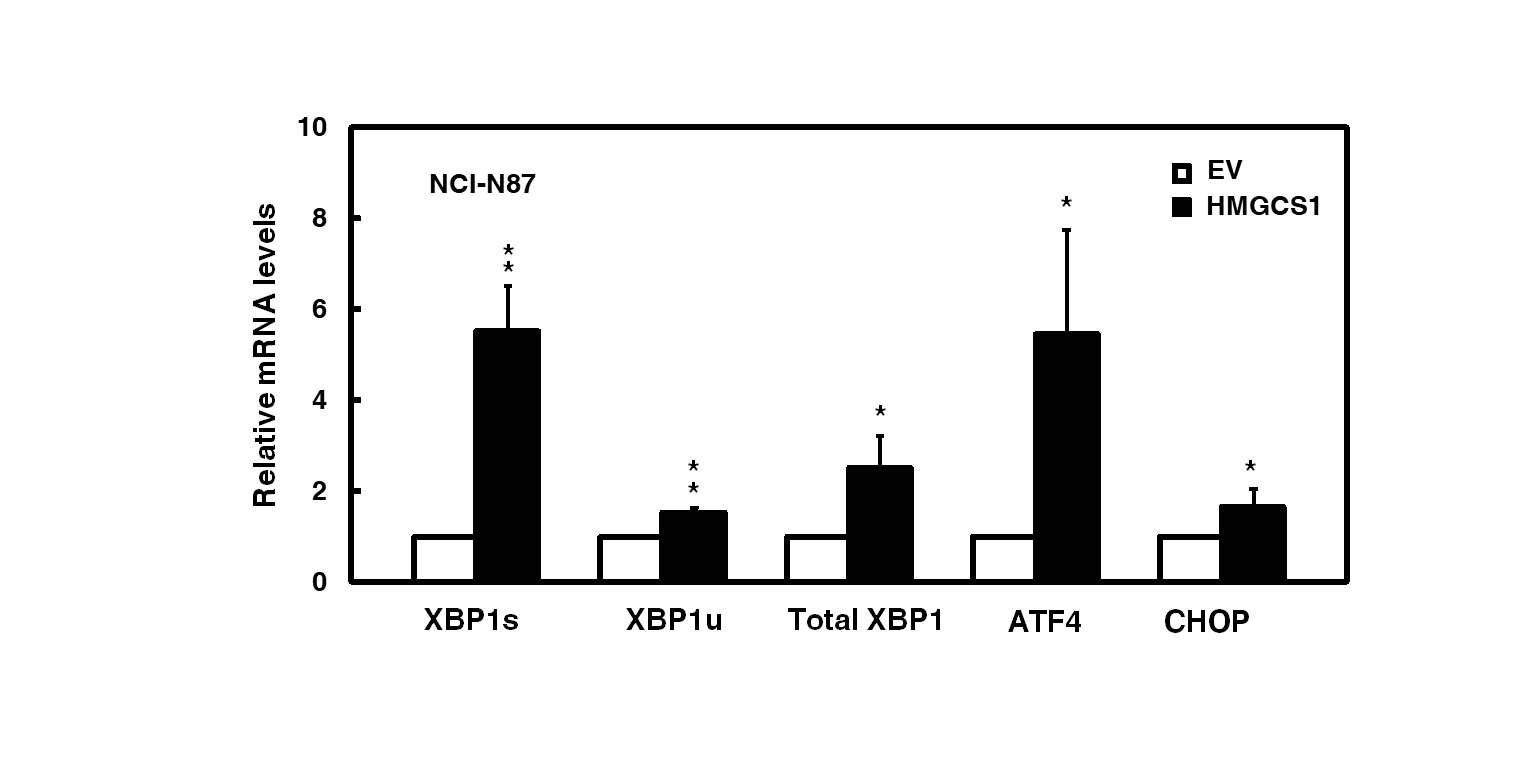


**Figure S9.** HMGCS1 enhances mRNA expressions of ER stress makers in NCI-N87 cells. NCI-N87 cells were transfected with HMGCS1-expressing construct (HMGCS1) or empty vector (EV) for 48 hours. The transcript levels of XBP1s, XBP1u, total XBP1, ATF4, and CHOP in the transfected cells were determined by quantitative real-time PCR and then normalized to GAPDH. Mean ± SD (*n* = 4). *, *P* < 0.05; **, *P* < 0.01.


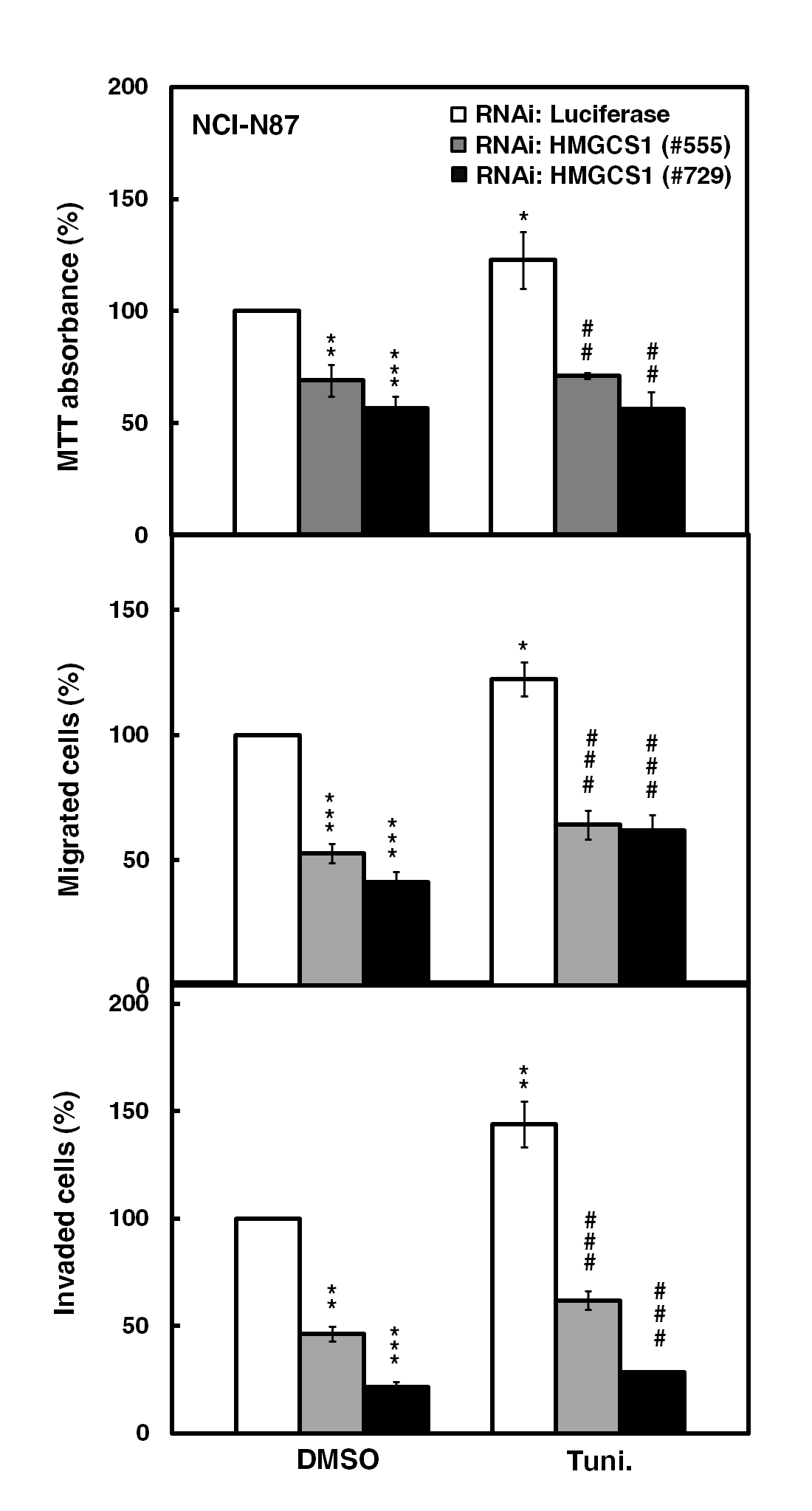


**Figure S10.** HMGCS1 knockdown inhibits tunicamycin-mediated increment of growth and progression abilities in NCI-N87 cells under mild ER stress. NCI-N87 cells were infected with lentiviruses expressing siRNAs against HMGCS1 (#555 and #729) or luciferase for 48 hours. Then the infected cells were seeded for the assays of MTT (*upper*), migration (*middle*), and invasion (*lower*). Cells were also treated with 2.5 μg/ml tunicamycin (Tuni.) or an equal volume of DMSO for 6 hours before assessment. Mean ± SD (*n* = 3). *, *P* < 0.05; **, *P* < 0.01; ***, *P* < 0.001. ##, *P* < 0.01; ###, *P* < 0.001.


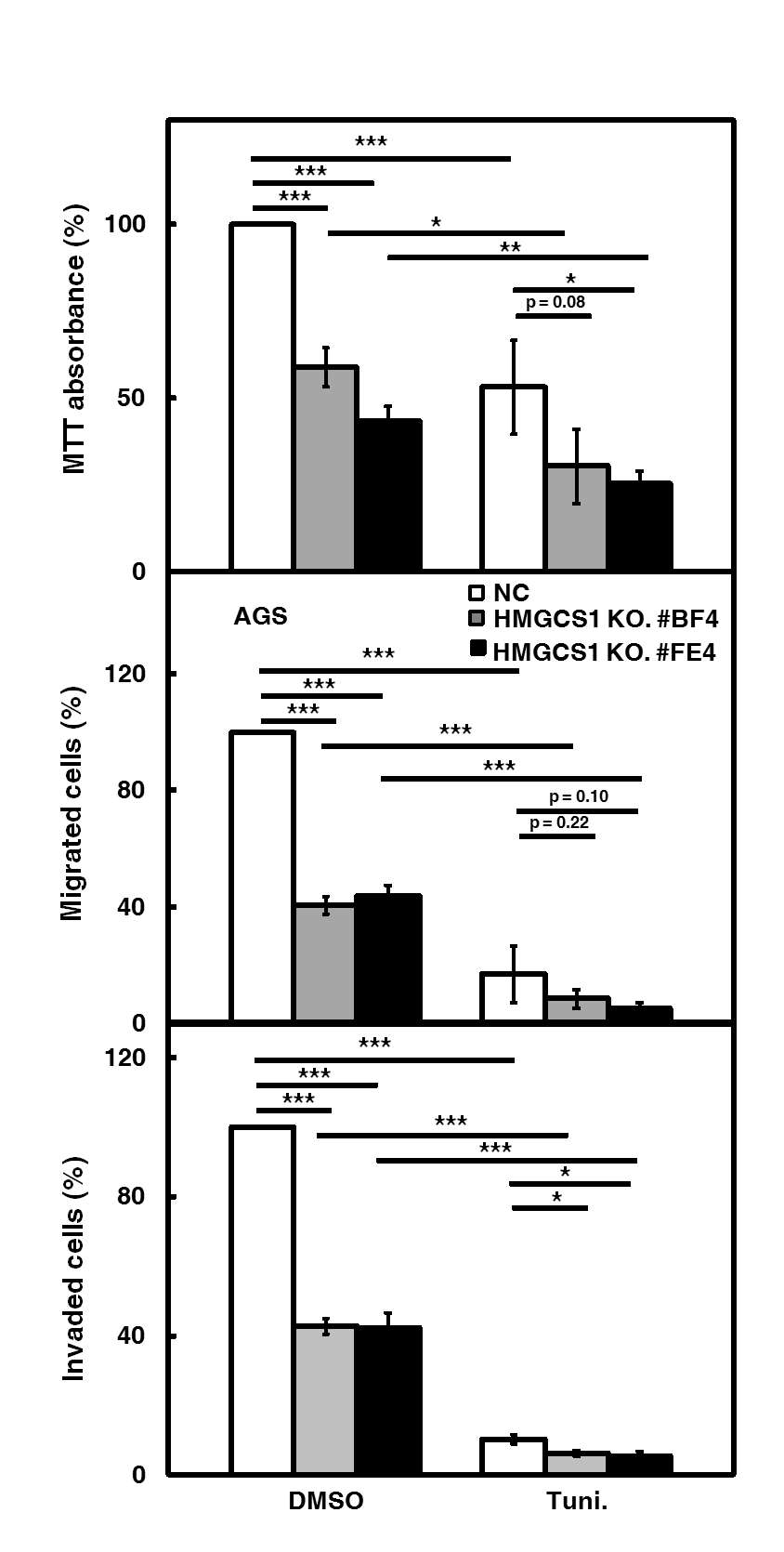


**Figure S11.** A longer tunicamycin treatment suppresses growth and progression abilities of AGS cells with and without HMGCS1 knockdown. HMGCS1 knockout (KO) #BF4 and #FE4 cells and negative control (NC) cells were seeded for the assays of MTT (*upper*), migration (*middle*), and invasion (*lower*) in the presence of 2.5 μg/ml tunicamycin or an equal volume of DMSO. Mean ± SD (*n* = 3). *, *P* < 0.05; **, *P* < 0.01; ***, *P* < 0.001.
